# Effect of water to cement ratio on mechanical properties of FRC subjected to elevated temperatures: Experimental and soft computing approaches

**DOI:** 10.1016/j.heliyon.2024.e39513

**Published:** 2024-10-18

**Authors:** M.S. Moradi, M.H. Tavana, M.R. Habibi, M. Amiri

**Affiliations:** Department of Civil Engineering, Kermanshah Branch, Islamic Azad University, Kermanshah, Iran

**Keywords:** Fiber reinforced concrete, Compressive strength, Tensile strength, Polypropylene fiber, Steel fiber, Machine learning

## Abstract

The deterioration of concrete is greatly dependent on the cracks and microcracks development owing to loading or environmental influences. An effective step to avoid the spread of cracks and microcracks is employing of different fibers in concrete and the manufacture of fiber reinforced concrete. On the other hand, fire is one of the cases that always threatens engineering structures. Elevated temperatures cause obvious chemical and physical changes that lead to concrete deterioration. In this study, the effect of adding various fiber with different volume contents in concrete mixture with various cement content was evaluated experimentally. Results indicate that spalling was more dominant in mixture containing steel fiber and with higher amount of cement, while there is not any spalling in mixture containing polypropylene (PP) fibers. Moreover, the reduction in tensile strength of fiber-free concrete specimens is less pronounced in mixtures with higher cement content. In addition, the positive performance of PP fibers compared to steel fibers was proved at higher temperatures and cement contents. Furthermore, by conducting a comprehensive and accurate survey, this research pinpoints gaps in the current literature. Moreover, the gathered dataset serves as input for training a machine learning approach known as Group Method of Data Handling (GMDH). The GMDH network demonstrates a satisfactory accuracy in predicting experimental results, with a MSE of 0.0044.

## 1 Introduction

In the past decades, numerous fires in engineering structures worldwide have been reported, posing a serious threat to both structural elements and the entire structure. The durability, physical, and mechanical aspects of mortar and concrete are significantly adversely impacted by exposure to fire. These effects result in substantial damage to structures, emphasizing the need to assess the performance of concrete at fire condition. High temperatures can induce spalling in concrete sections due to the vapor stress build up in pores and the non-uniform distribution of thermal pressures. This, in turn, can lead to critical collapses of structural elements [1], [2], [3]. The vapor pressure is without intermediaries linked to the free water content in the concrete matrix, or, in other words, the water to cement ratio (W/C). Additionally, exposure of concrete to elevated temperatures triggers chemical changes, including the disintegration of calcium hydroxide, decarbonation of carbonates, and disintegration of aggregates. These chemical transformations tend to weaken the concrete mechanical characteristics.

To mitigate the threat of high temperatures to concrete, incorporating fibers into the concrete mixture is a viable solution. The effectiveness of these fibers depends on various aspects, namely the matrix properties along with the mechanical properties (composition) and geometrical properties (size and shape) of the fibers [4]. Fibers made from carbon, polymers, steel, and glass are frequently employed in various application of fiber-reinforced concrete (FRC) [5]. Among those fibers, Polypropylene (PP) and steel stand out as cost-effective and widely used materials in the concrete industry [4]. Numerous studies have demonstrated that incorporating polypropylene fibers in concrete enhances its fire performance. These fibers, which melt at temperatures above 100 °C, can create channels that help lessen vapor pressure. This is particularly crucial since concrete's mechanical properties tend to deteriorate beyond 300 °C [1]. Consequently, polypropylene fibers can act as a fuse, preventing spalling, as recommended by European [6] and Australian [7] design guidelines for concrete in fire. To address potential adverse effects of embedded cracks resulting from pre-made channels, steel fibers can be introduced to enhance bond strength between the two sides of the channels through a dowel action effect. This approach aims to minimize any negative impact while optimizing the benefits of fiber reinforcement.

Fibers can be broadly restricted into two classes according to their concrete functions with respect to fire resistance. The first category, which includes carbon, steel, and basalt fibers, typically demonstrates excellent performance at ambient temperatures and enhanced thermal reliability at high temperatures. When these fibers are integrated into mortar or concrete, they enhance strength even in the presence of fire and effectively reduce cracks propagation and initiation, because of the bridging effect. Lower melting points can be observed in the other class of fibers, such as PP, Nylon, Polyethylene (PE) and PVA fibers. Upon increasing the temperature in FRC containing these fibers up to a specific limit, the fibers undergo melting, finally causes vaporization of free water. Therefore, the reduced internal vapor pressure contributes to the protection of the concrete microstructure [8].

Comprehensive investigations have explored the residual properties of FRC when subjected to high temperatures. However, uncertainties persist regarding its fire resistance, emphasizing the need for a thorough examination before considering its application in structures prone to fire incidents [9]. Assessing its importance relative to steel fiber-reinforced concrete (SFRC) proves challenging owing to the dispersed nature of study on natural and polymeric fibers. The insight of its effectiveness in mitigating spalling-induced damage and mechanical degradation at elevated temperatures is also deficient and intricate, given the differences in materials, mix design of concrete, and testing methodologies. Furthermore, results of research on the fire performance of FRC exhibit significant variations owing to variations in the cementitious matrix and types of fibers used. Notably, synthetic fibers such as steel and PP have demonstrated notable enhancements in spalling resistance at elevated temperatures conditions.

Evaluating the effect of W/C ratio and the proportion of mixed fibers on the mechanical characteristics of fire-exposed ready-mix concrete is one of the objectives of this research. The investigation into heat-induced spalling was conducted experimentally on mixes with varying fiber percentages and W/C ratios. Additionally, the study seeks to identify the optimal consumption of mixed fibers. Moreover, through the conducting a comprehensive and precise survey, this study identifies gaps in the existing literature. The experimental research was designed to address and bridge the gaps identified in the existing literature. With an enriched dataset, one can employ statistical approaches to analyze the gathered dataset. The gathered dataset encompasses various elements, including the mix design of concrete (water, cement, supplementary cementitious material (SCM), sand, gravel, and the amount of fiber used), fiber geometrical and mechanical characteristics (such as diameter, length, and tensile strength), and details about maximum temperature and heating rate. Two distinct fiber categories, namely steel and PP fibers, were collected across a broad range of temperature, specifically from 100 to 1200 °C. The target value in this study is the ratio of mechanical properties of FRC after exposure to high temperatures compared to its mechanical properties before heating. The mechanical properties considered for this ratio include both compressive and tensile strengths. Additionally, the collected dataset serves as input for training machine learning methods, with the study specifically employing the Group Method of Data Handling (GMDH) as one of the powerful machine learning techniques. By integrating experimental results with machine learning, the study aims to offer novel insights and predictive models that can guide the design of thermally resilient concrete mixes for practical applications in high-temperature environments.

## 2 Literature review

### 2.1 Spalling behavior

One effective strategy to enhance the durability of fire-exposed concrete involves incorporating fiber reinforcement. Numerous studies have verified that integrating randomly distributed fibers into concrete results in a notably enhancement in its both compressive and tensile strengths along with the ductility, and impact resistance under normal environmental conditions [2], [8], [9], [10]. FRC is widely utilized in construction for various applications such as tunnel linings, retaining structures, road and airport paving, precast panels, and ground-supported slabs. This is attributed to its outstanding capability to enhance the tensile strength of concrete and efficiently control the spread of microcracks and cracks. Concrete-based applications like those mentioned are susceptible to fire damage throughout their service life. Therefore, there has been an increasing interest in past decades in investigating the residual mechanical characteristics of fire-exposed concrete containing various types of fiber. To examine the residual mechanical properties of FRC using multiple types of fibers, numerous research studies have been performed. In the event of a fire, high-temperature exposure, or post-fire conditions, fibers in concrete contribute to several beneficial effects that enhance the residual mechanical characteristics of the material. The broad application of FRC in high-temperature settings faces challenges due to inconsistencies in global regulations and standards. Additionally, the complex thermal response of FRC, coupled with inherent uncertainties in its material properties, further complicates its adoption in such environments.

Steel stands as a primary material for fiber reinforcement, showcasing notable thermal conductivity and stability. It also exhibits crack-resistant properties that reduce stress due to thermal instability, particularly at elevated temperatures. These attributes stem from the effective heat transfer phenomena associated with steel fibers [11]. Steel fibers contribute reasonable tensile strength to mortar and concrete before, during, and after exposure to elevated temperatures, and they possess the capability to restraint microcracks through the fiber bridging effect. The effect of steel fibers on endurance of concrete to explosive spalling during elevated temperatures or fire exposure remains an area of uncertainty.

Numerous research [12], [13], [14] have indicated that incorporating steel fibers into concrete does not significantly reduce the risk of explosive spalling. In fact, Hertz [12] based on the experimental outcomes understood that the inclusion of steel fibers had not effect on mitigation the explosion risk, and notably, samples with a higher amount of steel fiber demonstrated a greater possibility of explosive spalling. In contrast, some investigations propose that steel fibers may aid in preventing explosive spalling in specimens subjected to elevated temperatures. This is attributed to the steel fibers' ability to lower the pressure inside pores in the matrix at elevated temperatures. Peng et al. [15] presented some results indicating steel fibers reduce the steam pressure build-up, while Bangi and Horiguchi [16] stated that including steel fibers enhances resistance to pore pressure in heated concrete.

Furthermore, the employing of steel fibers is shown to reduce the pressure inside pores in fire-exposed high-strength concrete [16], [17]. The effectiveness of steel fibers in lowering the pressure inside pores is notably greater during rapid heating compared to gradual heating, which can be associated to the higher thermal conductivity of steel fibers in relation to cement and aggregates. This property helps to minimize cracks caused by thermal gradients, allowing heat to be distributed more uniformly throughout the steel fiber-reinforced concrete. Moreover, research by Zheng et al. [18] and Gao et al. [19] indicates that the incorporation of steel fibers enhances transfer of heat within the matrix, causing a reduction in gradients of temperature and alleviating the impact of thermal shock at the surface. This ability of SFRC to diminish temperature gradients significantly contributes to its enhanced resistance against explosive spalling.

Fibers with lower melting points, such as PP, play a distinct role by creating tunnels for steam when they melt at specific temperatures, thereby reducing the likelihood of spalling. The primary goal of using these fibers is to diminish the risk of spalling and enhance fire response rather than providing structural response [20]. This mechanism was initially proposed by Khoury and Willoughby [21] to elucidate the functionality of PP fibers. The difference in polarity among PP fibers and the surrounding concrete matrix leads to weak interfacial bonding. This poorly bonded region, known as the Polypropylene-Infused Transition Zone (PITS), becomes vulnerable to disruption under elevated vapor pressure during high-temperature conditions. In this zone, micro-channels can form, allowing the movement of steam and serving as a pressure relief mechanism even before the melting of the fibers occurs.

### 2.2 Residual strength

**Figure 1:**
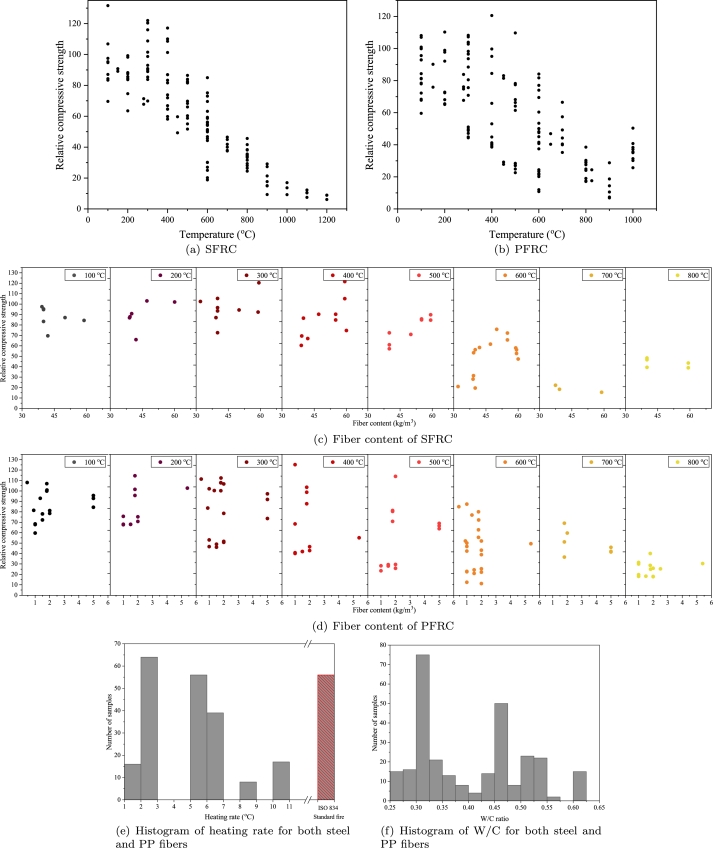
Results of the collected dataset.

Previous investigations on fire-exposed FRC have predominantly concentrated on FRC reinforced with PP and steel fibers. The compressive strength of SFRC after exposure to fire is significantly influenced by the exposure temperature and heating rate [22], [23], [24]. In general, there is a notable reduction in the relative compressive strength, denoted as $f_{c,20}/f_{c,T}$ where $f_{c,20}$ represents the compressive strength at ambient temperature and $f_{c,T}$ is the residual compressive strength of FRC after exposure to a temperature of T, as the temperature increases. Figs. 1(a) and 1(b) indicate the compressive strength of SFRC and PFRC after being exposed to high temperatures. As evident, the compressive strength of FRC reduces for various reasons, including thermal degradation of the cement paste, disparate thermal coefficients between the cement paste and fibers, and the accumulation of pore pressure.

Fig. 1(c) can be generated to evaluate the relative compressive strength under the impact of steel fiber volume. It is evident that a rise in fiber content positively influences the compressive strength of SFRC after being exposed to high temperatures, especially at higher temperatures, i.e., exceeding 300 °C. This observed improvement could be because of the bridging effect of fibers made of steel in the presence of microcracks and cracks as well as the positive impact of these fibers to improve the integrity of concrete in its deterioration stage. In this stage, the strength reduction may be attributed to various processes, including the transformation of CSH to *β*-C2S and C3S (beyond 560 °C), dehydroxylation of portlandite (450–550 °C), the conversion of *α*-quartz to *β*-quartz (at 573 °C), or the decarbonation of calcium carbonate (700–900 °C) [25]. In the deterioration stage, the presence of steel fibers hindered crack propagation and sustained the strength of the samples. Beyond 300 °C, strength reduction occurred owing to uneven shrinkage and expansion misalignment among the cement paste and aggregate. This is the rationale behind the advantageous effect of steel fibers in mitigating concrete deterioration [26].

Fig. 1(d) depicts the impact of amount of fiber on the compressive strength of PFRC after being exposed to high temperatures at various temperatures. Notably, there is no discernible consistent trend between fiber content and relative compressive strength. To the authors' best knowledge, there is no definitive conclusion regarding the impact of PP fiber content on relative compressive strength. While Zheng et al. [27] and Eidan et al. [28] dismiss the effect of PP fiber amount and volume on the relative compressive strength, Ju et al. [29] found a fiber amount of 8.1 $kg/m^{3}$ as optimal for enhancing the residual strength of PFRC. They conducted an analysis of PFRC compressive strength after exposure to a heat source with a temperature of around 350 °C. They concluded that the residual compressive strength of PFRC with 2.7 $kg/m^{3}$ PP was comparable to unreinforced concrete, suggesting that lower PP fiber content (less than 2.7 $kg/m^{3}$) is ineffective in enhancing strength up to 300 °C (with specimens exhibiting spalling beyond this temperature). In contrast, Hiremath and Yaragal [30] observed strength comparable to ambient temperature values even at a very low PP fiber content of 0.9 $kg/m^{3}$. Furthermore, a 8.1 $kg/m^{3}$ fiber content did not yield notable enhancement in residual strength up to 400 °C and exhibited a negative effect beyond the aforementioned temperature. They found that a PP fiber volume of 4.5 $kg/m^{3}$ is efficient in sustaining ideal residual strength. The significant disparity in their results may be caused by variations in other important factors. Hence, it is crucial to consider other parameters like aspect ratio, heating rate, concrete mix design, and so forth, to draw a conclusive understanding.

The mechanical characteristics of samples made by FRC can be influenced by different heating rates. Currently, researchers commonly employ two heating methods. One approach replicates real fire conditions by raising the temperature logarithmically to reach the target temperature, while the other involves a constant rate of temperature increase followed by maintenance at the target temperature. The latter method, often used for testing the spalling resistance of FRC materials, features a significantly faster temperature increase. The rapid temperature rise can create substantial temperature gradients, causing thermal damage to concrete and inducing varying thermal stresses in different regions. Additionally, higher heating rates can lead to the swift vaporization of moisture within the concrete matrix. The sudden release of vapor may generate internal pressure, resulting in spalling or cracking of the FRC. Previous studies in the literature have reported various heating rates ranging from 0.5 °C/min to 30 °C/min, with rates between 1 °C/min and 10 °C/min being the most commonly selected [31]. Fig. 1(e) illustrates a histogram of the diverse heating rates. Most of the existing data pertain to lower heating rates, specifically those below 7 °C/min. Nevertheless, there is a substantial number of tests conducted using ISO 834.

**Figure 2:**
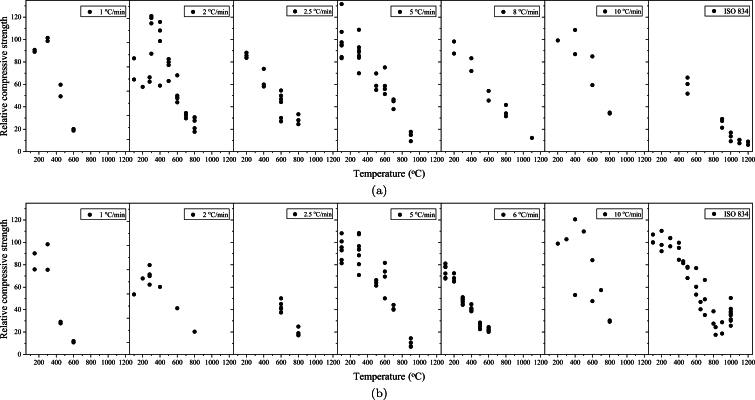
The influence of heating rate on relative compressive strength for a) steel fiber, and b) PP fiber.

Figs. 2(a) and 2(b) depict how relative compressive strength is influenced by both heating rate and elevated temperatures across steel and PP fiber. It is evident that increased temperatures lead to a decrease in relative compressive strength across various heating rates and fiber types. While higher heating rates can prompt swift and intense temperature rises within the concrete, potentially causing thermal stresses and cracking, it becomes apparent that the duration of the constant temperature time in which specimens are hold plays a more critical role [31]. Qadi and Zaidyeen [32] and Yu et al. [33] observed that an extended duration of heating time could lead to a more pronounced loss of strength. As the decomposition rate of calcium carbonate is gradual, an extension in heating duration results in increased degradation. Consequently, the ability of the section to sustain loads decreases at a later stage. Bangi and Horiguchi [16] found that rapid heating following the ISO 834 curve resulted in a similar pressure inside pores at a depth of 1 cm from the sample surface in comparison with a low heating rate of 5 °C/min. However, at a depth of 50 mm, the maximum pore pressure doubled due to the abundant water vapor created by an intense increase in temperature prior to capillary pores were evacuated. In contrast, Phan [34] presented opposing findings, indicating that the heating rate of 25 °C/min generated a smaller peak pore pressure than the heating rate of 5 °C/min. This difference was suggested to be linked to early microcracks presented by the greater heating rate. These conflicting results underscore the existence of other crucial parameters that have either been overlooked or not reported in previous studies. This emphasizes the necessity of considering a comprehensive set of factors that impact the response of fire-exposed FRC. For example, Xiong and Liew [35] noted that increasing the quantity of fibers can mitigate the variations in compressive behavior observed at different heating rates. Their study investigated the impact of heating rates (5 °C/min and 10 °C/min) on UHPC samples with three distinct PP fiber volumes (0.001, 0.0025, 0.005). It was concluded that at a small fiber content (0.1% of concrete volume), a greater heating rate resulted in a substantial reduction in Young modulus of elasticity and compressive strength. Nonetheless, as the fiber volume rised, the disparity in residual compressive strength between the two heating rates diminished.

**Figure 3:**
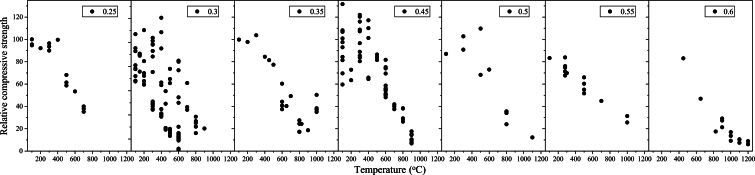
The influence of water to cement ratio on relative compressive strength for different fiber type.

Fig. 3 demonstrates the influence of the ratio of W/C on different fiber types at elevated temperatures. The data for a W/C ratio of 0.4 is not presented due to insufficient available data. It is notable that increasing heat exposure leads to a decline in relative compressive strength. However, this reduction is more pronounced in specimens with lower W/C ratios at temperatures more than 400 °C. Additionally, for lower W/C ratios, there is a notable decrease in the number of samples experiencing higher temperatures, indicating susceptibility to spalling. In these cases, reporting relative compressive strength becomes challenging as spalling is more likely to occur [31], [36]. Furthermore, concrete mixtures with lower W/C ratios exhibit superior performance at lower temperatures (i.e., below 400 °C) compared to samples with high W/C ratios. The reason of this difference can be associated to factors and parameters such as aggregate types and the quantity and quality of cementitious materials in specimens with relatively lower W/C ratios [37]. The steeper reduction in relative compressive strength for specimens with lower W/C ratios can be attributed to various factors, including increased volume of aggregates, transformation of calcium hydroxide to calcium oxide, reduction and disintegration of CSH, type of admixtures, and so on [38]. Therefore, the interdependence of these parameters to accurately identify their individual effects must be considered in future studies.

In summary, the fire resistance of FRC is a complex interplay of numerous factors, encompassing concrete mix design, aggregate types, fiber characteristics, heating rates, maximum temperatures, and more. The diversity in reported studies arises not only from the inherent variability in these influencing factors but also from the disparate heating and cooling methodologies employed. The obtained mechanical properties of FRC exhibit significant variations due to these methodological distinctions. It is important to adopt an integrative approach that concurrently considers all affecting parameters while acknowledging their intricate interactions. Such an approach is essential for comprehensively understanding and predicting the performance of FRC under elevated temperature conditions [39]. Presently, there is a notable absence of comprehensive models that encompass all critical factors, hindering the accurate prediction of mechanical properties in FRC subjected to high temperatures [25]. This underscores the need for further research and the development of comprehensive models to enhance our understanding of the behavior of FRC in fire-exposed conditions.

## 3 Experimental program

### 3.1 Material

**Figure 4:**
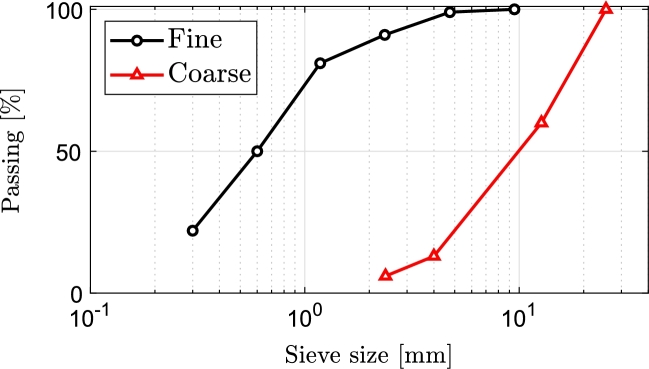
Grading curve of aggregates.

Fiber-reinforced concrete was manufactured using ordinary Portland Type-II cement, and the detailed specifications can be found in Table 1. All mixtures were composed of clean, well-graded natural aggregates, with coarse and fine aggregates having unit weights of 2.72 and 2.68, respectively. The grading curves of aggregates are shown in Fig. 4. The production and curing of all samples were carried out using potable water. To achieve the specified workability, the amount of superplasticizer, ranging from 1.0% to 1.6% of the cement weight, was determined through slump tests conducted in accordance with BS 1881 [40]. The recorded slump test results for the various concrete mixtures ranged between 45 and 115 mm. Each mixture was treated with Carboxal HF5000 high-range water reducer containing polycarboxylic acid–based.

**Table 1:** Various specification of the implemented Portland cement.

Chemical Specification	SiO_2_	Al_2_O_3_	Fe_2_O_3_	CaO	MgO	SO_3_	K_2_O	Na_2_O	L.O.I	I.R	Free CaO	C_3_S	C_2_S	C_3_A	C_4_AF
	20.7	5.2	4.6	65	1.8	2.2	0.5	0.15	1	0.4	1.3	59.5	14.5	6	14
Physical Specification	Specific surface [m/2kg]			Specific gravity [$kg/m^{3}$]			Initial Setting Time [min]			Final Setting Time [min]			28-day compressive strength [MPa]		
	320			3120			140			240			53

In the experimental process, two distinct types of fibers are employed: Polypropylene (PP) and steel (S) fibers. The study focuses specifically on steel and PP fibers because these are the most common and widely available fibers used in the construction industry. The mechanical and geometrical properties of these fibers can be found in Table 2. For concrete reinforcement, stainless steel fibers with 25 mm in length and 0.40 mm in diameter (aspect ratio = 62.5) were utilized. This particular steel fiber type is classified by a standard tensile strength (less than 1700 MPa), a melting point of around 1540 °C, and a density of 7870 kg/m^3^.

**Table 2:** The mechanical and geometrical properties of the fibers utilized in the tests.

Specification	Specific mass (kg/m^3^)	Length (mm)	Diameter (μm)	Melting temperature (°C)	Strain (%)	Tensile stress (MPa)	Modules of elasticity (GPa)
Polypropylene fiber (PP)	1000	18	23	165	8-10	400	4
Steel fiber (S)	7870	25	40	1538		1700	210

The experimental tests has been conducted on 27 concrete mixes: three of non-fiber reinforced concrete (350-0, 400-0, and 450-0) and 12 of steel fiber (S) and another 12 specimens of polypropylene fiber (PP) reinforced concrete.

### 3.2 Methods

As part of the study's objective, 24 distinct fiber-reinforced concrete mixtures have been created using two distinguish fiber types (oi.e., steel and PP) and four different volumetric proportions: 0.25%, 0.5%, 0.75%, and 1.0%. These fiber reinforced concrete specimens are subjected to four different temperatures, i.e., 20, 200, 400, and 600 °C. The 600 °C temperature limit is selected to ensure practical experimental feasibility and provide valuable insights into the performance of FRC under high-temperature stress. At around 600 °C, concrete experiences significant property changes, including the onset of calcium hydroxide dehydration, a drop in mechanical strength, and the development of stresses due to thermal gradient [41]. Setting this temperature threshold allows for an effective evaluation of how various fibers, such as steel and PP, reduce these effects and help maintain the structural integrity of concrete elements under extreme conditions.

**Figure 5:**
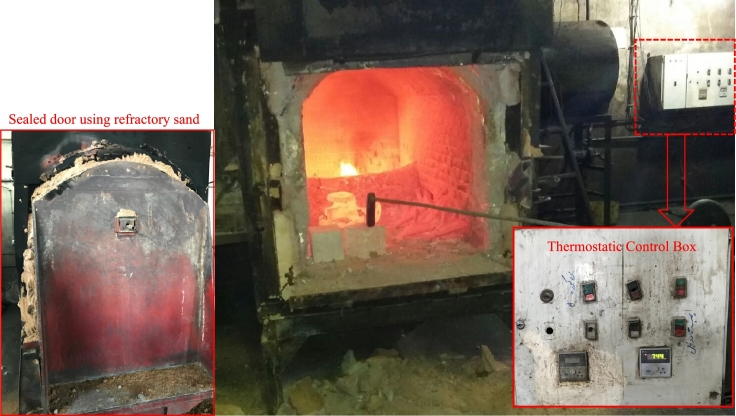
Furnace and control box used in the experiment.

The proportion of the concrete mixtures are summarized in Table 3. The compression and tensile experiments on the samples were conducted according to principles suggested in ACI 318-19 [42]. The procedure suggested in ASTM C192 was used to mix materials [43]. The consumed concrete was ordered from a local supplier. Non-fiber concrete was transported to the experimental site, and fibers were incorporated into the concrete as per their specified mix design. The concrete and fibers were put into the mixer, undergoing a mixing process for approximately 2 minutes to guarantee the uniform distribution of fibers within the mixture. Then, the molds were filled up and were put in the humid room with the temperature of 22±3 °C for 24h and taken away after that, and the samples were kept in potable water for 7 and 28 days for curing and conducting the subsequent compression and tensile tests along with fire tests. Four distinguished temperatures were applied to the specimens based on the objective of the study. For normal conditions and elevated temperatures, the specimens are taken from water after the specified days and wiped out. A gas furnace was used to heat the samples. The heat applied to the samples was indirect, and this heat could be programmed, controlled, and monitored by a digital control box installed next to the furnace (Fig. 5). After placing the concrete samples inside the furnace, the door was completely sealed with refractory sand to lessen temperature exchange with the surrounding environment and to enhance the furnace's efficiency and performance.

**Table 3:** The amount of material used to make different concrete mixtures (All units are in *kg*/*m*^3^).

Concrete type	350	400	450
Cement	350	400	450
Water	195	197	180
Fine aggregates	1084	1055	1052
Coarse aggregates	735	645	722

All samples in the furnace were heated at an escalating rate of 10 °C per minute and then maintained at the target temperature for 1 hour. This heating rate was chosen as a compromise, reflecting the rates found in typical fires, which are estimated to be between 10 and 20 °C/min [19], [44]. Once the specimens were heated, they were allowed to cool in ambient temperature, after which both the residual compressive and tensile strength of the samples were measured. For every test conducted, the reported outcomes represent the average of three samples. A total of 81 sets, each comprising three concrete specimens (54 sets designated for compressive tests and 27 sets for tensile tests), underwent exposure to three distinct maximum temperature levels. This comparative analysis aimed to ascertain the impact of elevated temperatures on the reduction of mechanical characteristics.

## 4 Results and discussion

### 4.1 Compressive strength

**Figure 6:**
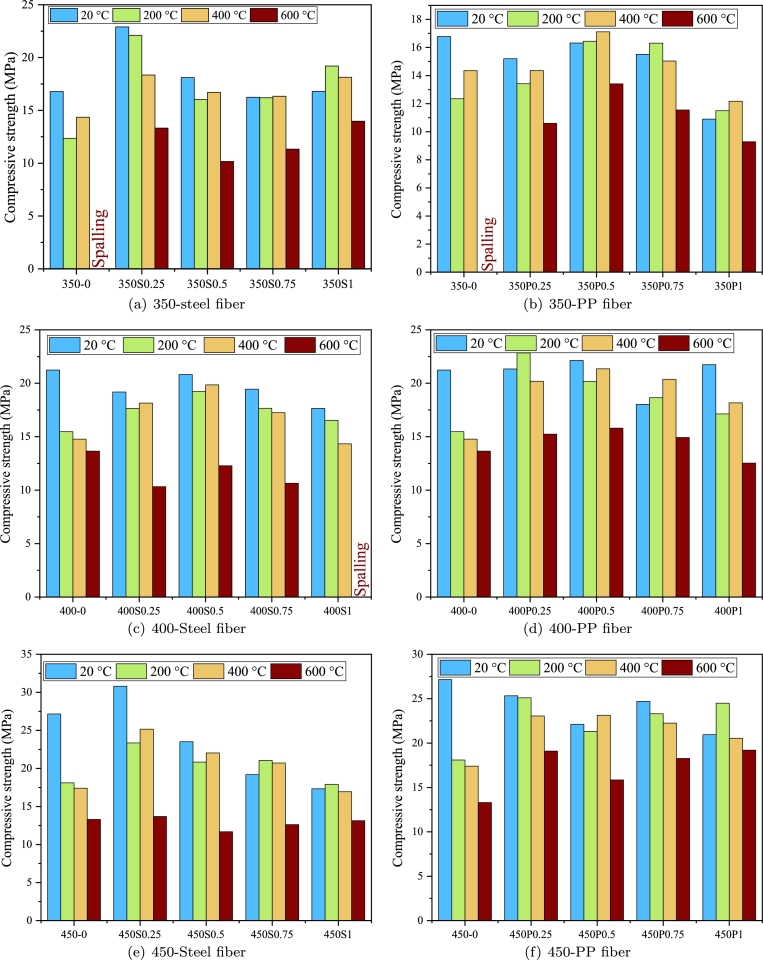
The experimental results of 7-day compressive strength.

**Figure 7:**
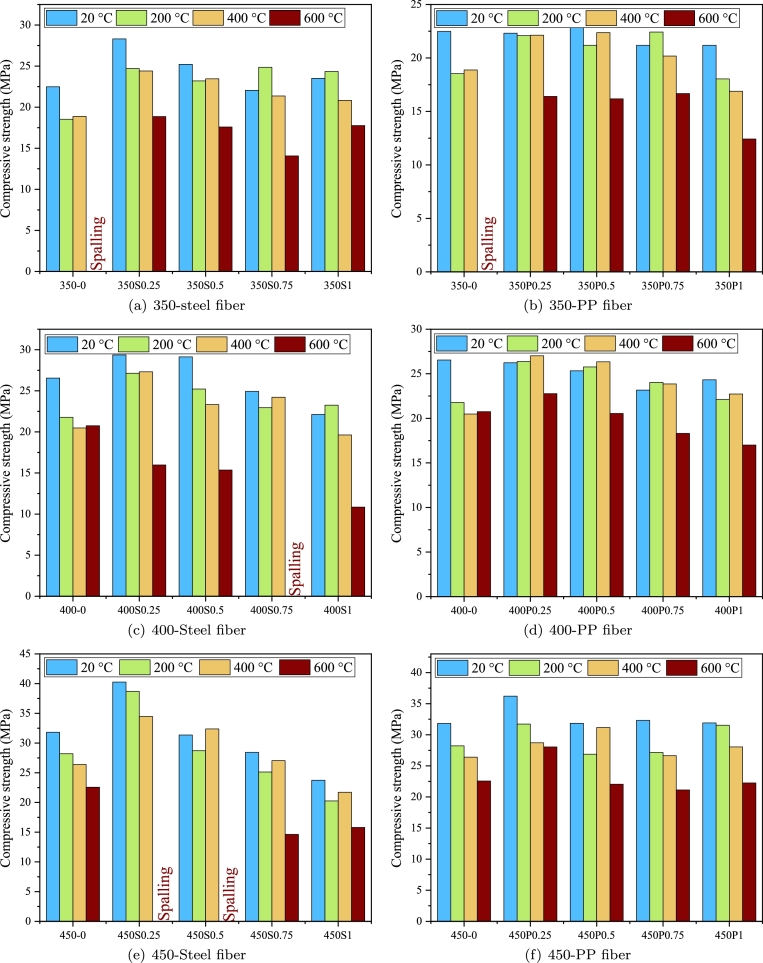
The experimental results of 28-day compressive strength.

Table 4 summarizes the compressive strength results for concrete specimens at various ages and temperatures. For a clearer representation of the findings, Figure 6, Figure 7 have been generated. Generally, the compressive strength of concrete at both ages decreases with rising temperatures. The reason for observed decline may be associated to chemical and/or physical changes, including free water evaporation, dehydration, aggregate expansion, and the formation of micro-cracks [1]. Furthermore, some mixtures experience a destructive failure known as spalling. Thermal stresses throughout the specimen and vapor stress inside micro holes are two primary causes of destructive failure, i.e., spalling, in mixtures exposed to elevated temperatures. This phenomenon is more prominent in mixtures containing steel fiber and a higher amount of cement. The dense structure of mixtures with a higher cement content slows down the escape of vapor from the specimen, leading to a significant increase in pore pressure and resulting in spalling failure. Fibers with lower melting temperatures may provide a pathway for vapor escape, preventing spalling. As a result, mixtures containing PP fibers do not exhibit spalling behavior.

**Table 4:** The compressive strength of specimen at elevated temperature [units are in MPa].

	20 °C		200 °C		400 °C		600 °C	
Mixture	7-day	28-day	7-day	28-day	7-day	28-day	7-day	28-day
350-0	16.78	22.48	12.35	18.53	14.35	18.88	Spalling	Spalling
350S0.25	22.90	28.31	22.10	24.71	18.35	24.40	13.33	18.86
350S0.5	18.11	25.21	16.02	23.20	16.71	23.45	10.17	17.59
350S0.75	16.24	22.05	16.20	24.85	16.34	21.36	11.33	14.07
350S1	16.80	23.51	19.20	24.35	18.14	20.84	13.98	17.77
350P0.25	15.20	22.31	13.42	22.08	14.36	22.13	10.60	16.40
350P0.5	16.32	22.91	16.44	21.18	17.12	22.37	13.41	16.18
350P0.75	15.52	21.18	16.31	22.42	15.04	20.18	11.55	16.67
350P1	10.90	21.18	11.50	18.04	12.17	16.88	9.29	12.42
400-0	21.24	26.55	15.47	21.77	14.78	20.48	13.65	20.75
400S0.25	19.18	29.37	17.65	27.14	18.15	27.32	10.33	15.97
400S0.5	20.82	29.14	19.24	25.22	19.85	23.33	12.28	15.36
400S0.75	19.45	24.94	17.66	22.95	17.25	24.22	10.65	Spalling
400S1	17.65	22.12	16.54	23.25	14.33	19.63	Spalling	10.85
400P0.25	21.34	26.24	22.84	26.38	20.19	27.01	15.24	22.77
400P0.5	22.14	25.33	20.17	25.77	21.35	26.34	15.80	20.55
400P0.75	18.03	23.17	18.65	24.03	20.37	23.86	14.93	18.31
400P1	21.74	24.32	17.13	22.12	18.17	22.73	12.53	17.00
450-0	27.16	31.82	18.1	28.22	17.4	26.4	13.3	22.57
450S0.25	30.80	40.26	23.35	38.70	25.16	34.48	13.69	Spalling
450S0.5	23.51	31.36	20.84	28.71	22.03	32.38	11.68	Spalling
450S0.75	19.18	28.44	21.05	25.14	20.72	27.04	12.62	14.63
450S1	17.33	23.73	17.91	20.26	16.95	21.73	13.13	15.81
450P0.25	25.33	36.22	25.10	31.73	23.05	28.71	19.10	28.04
450P0.5	22.11	31.85	21.33	26.88	23.14	31.17	15.85	22.05
450P0.75	24.70	32.32	23.31	27.15	22.25	26.65	18.27	21.14
450P1	20.95	31.91	24.49	31.55	20.54	28.04	19.20	22.26

A comparatively smaller heating rate applied to specimens in the current study can also contribute to spalling occurrences. Research suggests that higher heating rates have a beneficial effect on mitigating spalling in concrete. When concrete is heated at a higher rate, it develops more surface cracks. These cracks can serve as pathways to escape the vapor, thereby mitigating internal stress and the likelihood of spalling [34], [45]. To investigate the effect of heating rate, Felicetti et al. [46] examined four different heating rates—1, 2, 10, and 120 °C/min. Their study revealed that while higher heating rates lead to higher peak pressures, the resulting thermal cracks increase the concrete's permeability and facilitate pressure release. Similarly, Phan [34] found that a higher heating rate promotes the formation of micro-cracks, which helps to alleviate internal pressure. Consequently, fire-induced cracks can effectively aid in reducing internal pressure, thereby lowering the probability of spalling.

Fig. 6(a) demonstrates incorporating of steel fibers into specimens with a binder amount of 350 $kg/m^{3}$ at 7 days and at room temperature leads to a notable enhancement in mechanical properties of concrete in terms of compressive strength, with a mean rise of 10.3%. However, this improvement diminishes with further increases in fiber content, indicating a potential threshold beyond which additional fibers may not contribute positively to the material's performance. This trend is consistent with the observed 28-day compressive strength of FRC specimens containing the exact cement content, suggesting that the early-age gains in strength may be retained or even slightly improved over time under optimal fiber proportions.

In contrast, FRC samples containing PP fibers exhibit a reverse trend, resulting in average decreases in compressive strength by 13.7% at 7 days (Fig. 6(b)) and 2.6% at 28 days (Fig. 7(b)). The reduction in strength may be due to the intrinsic mechanical properties of the PP fibers, which, because of their lower yield stress and modulus of elasticity, and smaller diameter, are less effective in elevating the load-bearing capacity of the concrete matrix [47], [48], [49]. Unlike steel fibers, PP fibers lack the necessary stiffness and strength to effectively arrest crack propagation under compressive loads, leading to a degradation in overall performance [50], [51].

The superior performance of steel fibers is further reinforced by their ability to refine the bond strength among the fiber and the cement matrix. This is largely due to their increased length and diameter, which enhance mechanical interlocking within the concrete [50]. This stronger bond not only improves the material's capacity to bridge cracks but also contributes to the post-cracking strength of the concrete after being exposed to high temperatures [52], [53]. The arresting effect of steel fibers plays a crucial role in this context, as it limits the propagation of micro-cracks and delays the onset of macro-cracking, thereby preserving the integrity of the concrete at applied stresses. Moreover, the enhanced bond strength and crack-bridging ability of steel fibers contribute to a more ductile failure mode, which is desirable in structural applications where energy absorption and resistance to catastrophic failure are critical [54]. These findings align with previous research indicating that steel fibers, due to their robust mechanical properties and their synergistic interaction with the cement matrix, can significantly enhance the mechanical performance and durability of FRC, particularly in applications subjected to dynamic or cyclic loading conditions [55], [56].

The 7-day compressive strength of SFRC samples with cement contents of 400 and 450 $kg/m^{3}$ shows a notable reduction, averaging 10% and 17%, respectively (Figs. 6(c) and 6(e)). The same trend can be seen in mixtures containing PP fibers at 7 days with 400 and 450 $kg/m^{3}$ cement content at Figs. 6(d) and 6(f), respectively. This decrease in early-age strength can be primarily attributed to several factors related to the concrete mix design and the interaction between steel fibers and the cement matrix. One of the main reasons for this strength reduction is the insufficient water content in the concrete, which is crucial for the hydration process of cement [57]. In mixes with higher cement content, the available water may not be adequate to fully hydrate all the cement particles, leading to a less dense and weaker matrix. Additionally, the presence of steel fibers can exacerbate this issue by increasing the surface area that requires wetting, further straining the available water resources [58]. This insufficient hydration results in a less effective bond between the cement matrix and the fibers, reducing the overall strength of the composite material. Another critical factor is the potential for fiber clustering or agglomeration within the concrete mix. As noted by Papachristoforou et al. [59], when steel fibers are not uniformly distributed, they can create localized weaknesses or voids within the matrix. These clusters of fibers do not contribute effectively to load-bearing and can act as stress concentrators, leading to a reduction in compressive strength. This clustering effect is particularly problematic in high-fiber-content mixes, where the likelihood of fibers overlapping and creating dense clusters increases.

In contrast, cylindrical samples with lower fiber ratios exhibit an improvement in compressive strength, as shown in Table 4 and Figs. 7(a) and 7(b). This suggests that at lower concentrations, steel fibers are more likely to be well-distributed throughout the matrix, enhancing the material's load-bearing capacity by effectively bridging micro-cracks and contributing to a more homogeneous structure. A similar trend, though less pronounced, is observed in the 28-day compressive strength of these samples. Over time, the ongoing hydration process of the cement particles results in a complete and more compact cement matrix, which helps to mitigate some of the adverse effects caused by steel fibers [60]. The continued hydration allows for better bonding between the fibers and the cement matrix, which can compensate for the initial weaknesses observed at 7 days. This enhanced hydration process is particularly beneficial in the later stages of curing, where the development of further C-S-H gel contributes to increased strength and durability [61]. The 28-day compressive strength reduction rate for samples containing steel fibers with cement contents of 400 and 450 $kg/m^{3}$ is significantly lower, averaging 0.6% and 2.8%, respectively (Figs. 7(c) and 7(e)). This reduction is less severe compared to the 7-day results, reflecting the favorable effect of prolonged curing on the material's mechanical properties. The decreased rate of strength reduction at 28 days suggests that while steel fibers may initially disrupt the matrix, the long-term benefits of fiber reinforcement, such as improved toughness and resistance to cracking, begin to outweigh the initial drawbacks associated with fiber clustering and insufficient water content.

As the temperature applied to the FRC samples increased, the observation showed a notable reduction in the compressive strength. However, the incorporation of fibers significantly mitigated this reduction. Specifically, when different amounts of steel fibers were added to concrete mixes with cement contents of 350, 400, and 450 $kg/m^{3}$, the 7-day relative compressive strength at 400 °C increased by 21.1%, 17.7%, and 21.9%, respectively. Similarly, for PP fibers, the compressive strength at 400 °C improved by 2.2%, 35.4%, and 27.8% across the same cement contents, demonstrating a surprisingly superior performance of PP fibers at elevated temperatures compared to steel fibers (Figs. 7(d) and 7(f)). This enhanced performance of PP fibers can be attributed to their lower melting point, which creates micro-voids within the concrete matrix as the fibers melt. These voids may facilitate internal steam curing, where the generated steam promotes additional hydration of the cement, potentially resulting in a rise in compressive strength despite the high temperatures [9].

**Figure 8:**
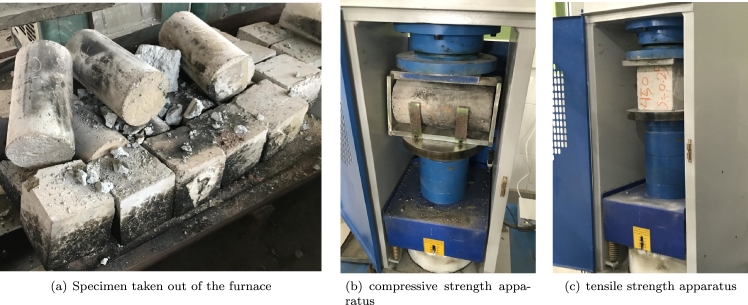
Spalling failure of FRC specimens along with the compressive and tensile test apparatus.

By increasing the temperature to 600 °C, certain FRC samples experienced spalling and explosive failures upon exiting the furnace. This phenomenon is primarily due to the buildup of water vapor within the concrete matrix. When the temperature rises rapidly, trapped water vapor within the pores of the concrete becomes pressurized, and if there is no escape route, this pressure can cause an abrupt and violent destruction of the material, known as spalling. This failure mode, illustrated in Fig. 8(a), was particularly evident in 350-0 samples at both 7 and 28 days, as well as in 450S0.75 at 28 days, 450S1 at 7 days, and 450S0.25 and 450S0.5 samples at 28 days. Notably, spalling was not observed in specimens containing PP fibers, underscoring a significant advantage of these fibers in preventing such catastrophic failures. The mechanism by which PP fibers prevent spalling lies in their thermoplastic nature; as the temperature rises, PP fibers melt and create micro-channels within the concrete. These channels act as micro tunnels for water steam to escape, thereby results in a reduction in the internal pore pressure and preventing spalling [1]. This self-regulating property of PP fibers under high temperatures enhances the thermal resilience of the concrete, making it particularly suitable for applications where fire resistance and durability are critical considerations [62]. The compressive and tensile test apparatus are shown in Figs. 8(b) and 8(c).

Subjecting concrete samples with 350 $kg/m^{3}$ cement content and containing steel fibers to 600 °C at the age of 7 days resulted in a 20.7% reduction in the compressive strength compared to the control specimen at room temperature. This reduction was 18.8% and 4% for samples with 400 and 450 $kg/m^{3}$ cement content, respectively. Significantly, the effectiveness of steel fibers at high temperatures is more prominent in higher cement content, attributed to a better arresting effect of fibers in a rich cement-based mixture [63]. Similarly, PP fibers exhibit a comparable function, and their efficiency at high temperatures seems to be unrestricted to the amount of cement used in concrete. The average rise in the 7-day compressive strength for PP-reinforced samples with 400 and 450 $kg/m^{3}$ cement content, compared to the heated control specimen at 600 °C, was 7.0% and 36%, respectively. It has been proposed that a higher amount of PP fibers can enhance crack control by distributing stress more equally, preventing propagation of cracks, and reducing their width [64]. However, a surplus amount of fiber can result in a higher porosity and poor distribution of fibers in the concrete mixture, which can adversely impact the bonding between cement matrix and fibers [9]. It is important to emphasize that finding the optimal fiber amount for attaining an ideal balance between beneficial and detrimental effects relies on various interconnected factors, such as the concrete mix design and the mechanical and geometrical properties of the fibers.

### 4.2 Tensile strength

**Figure 9:**
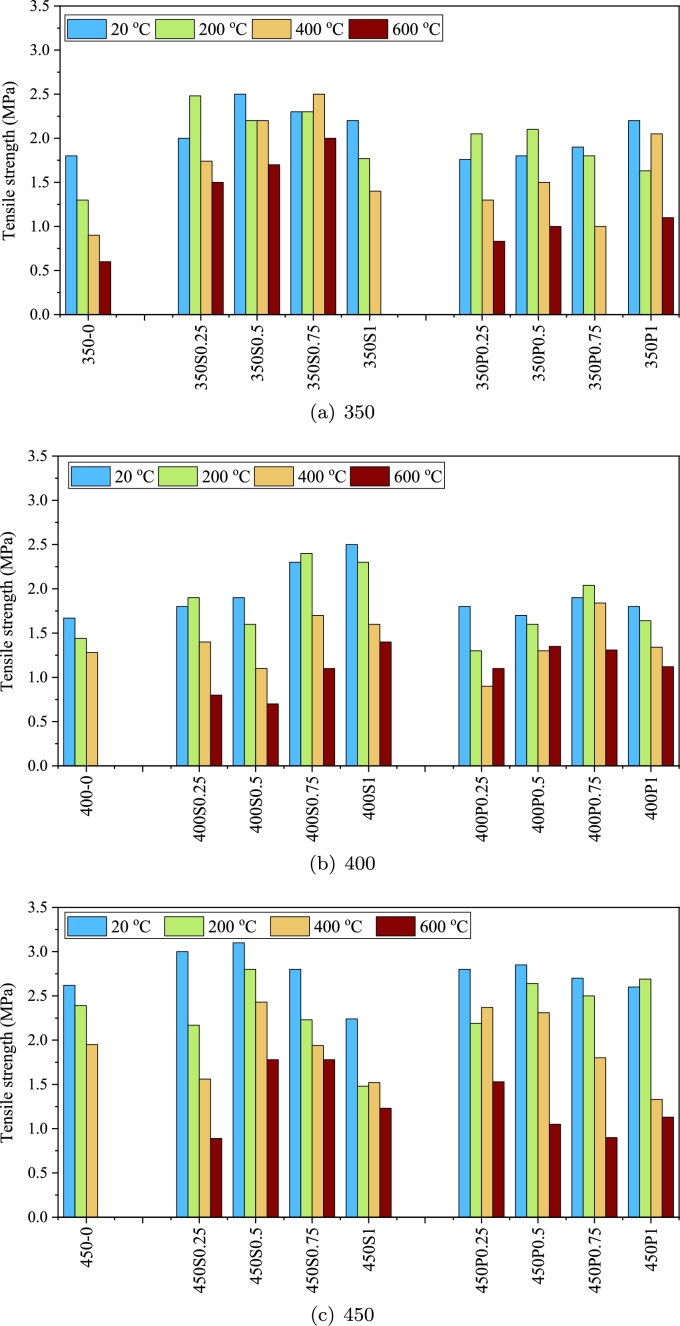
The experimental results of the tensile strength.

Splitting tensile strength tests were conducted to assess the effect of cement content, fiber type, fiber volume, and elevated temperatures on the tensile strength of FRC specimens. Figs. 9(a), 9(b), and 9(c) along with Table 5 illustrate the tensile strength of samples with diverse cement content. As the results indicate, increasing the temperature generally results in a reduction in the tensile strength of the samples. The diminished strength in specimens without fibers can be attributed to chemical changes in the concrete. Concrete is a heterogeneous material comprising cement paste, aggregates, and sometimes, steel bars or fibers as reinforcing elements. Understanding the response of each of these components to elevated temperatures is challenging and becomes even more intricate when combined as a structural member. According to research by Mindess et al. [65], cement paste contains several chemical components that exhibit different responses under elevated temperatures. Hydrated calcium silicate (C-S-H), constituting more than 50% of the cement paste volume, is one of the most crucial components. Additionally, calcium hydroxide (CH) typically accounts for 20 to 25% of the volume of the cement matrix. Water, acting as a residual element in cement paste and playing a vital role in the deterioration of resistance at high temperatures, is also present. The water added to the cement paste occupies the pores of the concrete, which generally fall into two categories: capillary cavities and cavities in cement gel. Capillary cavities have sizes ranging between 10 and 1000 *nm*, while the cavities in cement gel are less than or equal to 10 *nm*. The water in these cavities is distinct; evaporative water is found in capillary pores, while the water in gel cavities is considered part of C-S-H.

**Table 5:** The tensile strength of specimen at high temperatures [units are in MPa].

T (C)	20 °C	200 °C	400 °C	600 °C
350-0	1.8	1.3	0.9	0.6
350S0.25	2	2.48	1.74	1.5
350S0.5	2.5	2.2	2.2	1.7
350S0.75	2.3	2.3	2.5	2
350S1	2.2	1.77	1.4	Spalling
350P0.25	1.76	2.05	1.3	0.83
350P0.5	1.8	2.1	1.5	1
350P0.75	1.9	1.8	1	Spalling
350P1	2.2	1.63	2.05	1.1
400-0	1.67	1.44	1.28	Spalling
400S0.25	1.8	1.9	1.4	0.8
400S0.5	1.9	1.6	1.1	0.7
400S0.75	2.3	2.4	1.7	1.1
400S1	2.5	2.3	1.6	1.4
400P0.25	1.8	1.3	0.9	1.1
400P0.5	1.7	1.6	1.3	1.35
400P0.75	1.9	2.04	1.84	1.31
400P1	1.8	1.64	1.34	1.12
450-0	2.62	2.39	1.95	Spalling
450S0.25	3	2.17	1.56	0.89
450S0.5	3.1	2.8	2.43	1.78
450S0.75	2.8	2.23	1.94	1.78
450S1	2.24	1.48	1.52	1.23
450P0.25	2.8	2.19	2.37	1.53
450P0.5	2.85	2.64	2.31	1.05
450P0.75	2.7	2.5	1.8	0.9
450P1	2.6	2.69	1.33	1.13

Owing to the complexity of concrete constituents, determining the exact temperature at which concrete undergoes behavioral changes is challenging. However, various researchers, such as Fletcher et al. [66] and Hertz [67], have proposed an overall concrete response to elevated temperatures.

When the concrete mixture reaches a temperature of 100 to 140 °C, the water in the concrete begins to evaporate, leading to a rise in the internal stress of the mixture. In a situation that the temperature reaches 150 °C, the water in C-S-H evaporates, resulting in dehydrated cement paste and reduced volume that consequently, internal tensions increase [68]. At temperatures above 300 °C, these internal stresses cause small cracks, resulting in irreversible deformations. At 400 to 600 °C, the CH in the cement paste decomposes to produce calcium oxide and water steam. This decomposition significantly reduces the strength of concrete. At temperatures of 575 to 800 °C, decrease in strength begins owing to chemical changes in the aggregates. Quartz-based aggregates increase in volume at around 570 °C and calcareous aggregates decompose at 800 °C, at which temperature the concrete will turn into an aggregate mass. For this reason, the Eurocode does not include any strength for concrete beyond 600 °C, and in theory, concrete has no bearing resistance at this temperature [6].

The average reduction in tensile strength in specimens without fibers at 350, 400, and 450 cement content for different temperatures are 48.3%, 18.6%, and 17.2%, respectively. The reduction in tensile strength of fiber-free concrete samples is less pronounced in groups with higher cement content. The reason for this can be associated to the greater amount of cement and the greater and better progress of the hydration process, which makes the cement paste more integrated. However, the tensile strength of samples without fiber is very low. On the other hand, with increasing temperature, the tensile strength of samples without fibers containing lower cement content reduces at a higher rate.

In some specimens, the tensile strength increased compared to the control sample after exposing to elevated temperatures. This was especially the case in samples exposed to 200 °C. This observation can be ascribed to high-temperature curing, which increases the cement hydration process [27]. Additionally, the increase in temperature may enhance Van der Waals forces by removing absorbed water, hence leading to a rise in compressive strength [69]. Similar results were seen in other studies [15], [70], [71], [72], [73].

As the temperature increases, a corresponding reduction in the tensile strength of FRC samples is typically observed. However, the inclusion of fibers significantly enhances the tensile strength of the cylindrical specimens, effectively mitigating the unfavorable impacts of high temperatures [74]. For instance, specimens containing steel fibers with a cement content of 350 $kg/m^{3}$ exhibited an average tensile strength increase of 68.2% at 200 °C. The tensile strength improvement becomes more pronounced as the fiber volume in the concrete increases, with steel fiber-reinforced specimens showing tensile strength enhancements of 117% at 400 °C and an impressive 188% at 600 °C. This indicates that steel fibers are particularly effective in maintaining and even enhancing tensile strength at high temperatures, especially in mixes with lower cement content where the matrix might be less dense. The effectiveness of fiber reinforcement is not limited to steel fibers; polypropylene fibers also contribute to tensile strength improvements, with increases of 45.7%, 62.5%, and 62.7% observed at 200 °C, 400 °C, and 600 °C, respectively. The performance of PP fibers at elevated temperatures is referred to their ability to melt and create a network that helps in stress redistribution and crack bridging, although their impact is slightly less pronounced than that of steel fibers.

By increasing the cement content to 400 $kg/m^{3}$, the efficiency of steel fibers at different temperatures in improving the tensile strength are 42.3, 13.2, and 66.6%, respectively. The improvement of tensile strength due to utilization of PP fibers is equal to 22.2%, 16.6%, and 110%, respectively. A higher amount of cement content weakens the performance of steel fibers at elevated temperatures so that adding fibers at temperatures of 200 and 400 °C reduces the tensile strength by an average of 10 and 4.5%, respectively. However, PP fibers in these two temperatures increased by 4.8 and 0.1%, respectively, which indicates the positive performance of PP fibers compared to steel fibers at higher temperatures and cement contents. The changes that concrete withstands at high temperatures are in the micro-scale, which affect both concrete and steel fibers. Owing to the differences in the steel fiber and concrete chemical composition, there are differences in the thermal properties of the whole complex. Therefore, internal stresses are created, leading to cracks and loss of adhesion between concrete and steel. This means that the properties of concrete and steel materials are reduced by heat [66]. Higher cement content in the concrete leads to a denser environment which makes water vapor can not escape, and this will cause the concrete to spalling or create cracks in the element.

## 5 Soft computing method

Utilizing soft computing methods offers the advantage of enhancing the usability and accuracy of models when handling complicated natural systems characterized by a multitude of influencing parameters [39]. Machine learning, particularly the Group Method of Data Handling (GMDH), exemplifies one such soft computing model. GMDH is an inductive modeling approach employed for tasks like data analysis, prediction, and optimization. This algorithm operates as a self-organizing method, constructing a series of polynomial models that incrementally increase in complexity to approximate a given dataset. It commences with simple models and progressively advances to more intricate ones, selecting the best-performing models at each stage. The selection criteria are based on factors such as accuracy, error minimization, or other performance metrics. A notable feature of GMDH is that the manifold and structure of the network are minimally impacted by human interference, with the network's configuration gradually self-organizing. The connections in the input toward output features are delineated by the Volterra polynomial series (Equation (1)).

(1) $$\overset{¯}{y}=w_{0}+\sum_{i=1}^{n}w_{i}x_{i}+\sum_{i=1}^{n}\sum_{j=1}^{n}w_{ij}x_{i}x_{j}+\sum_{i=1}^{n}\sum_{j=1}^{n}\sum_{k=1}^{n}w_{ijk}x_{i}x_{j}x_{k}+...$$

In the context of the GMDH model, where *x* represents the vector of input parameters, *w* denotes the vector of weights or parameters, *n* stands for number of input features, and the bar sign indicates the predicted outcomes, the application of the GMDH method involves utilizing a partial quadratic polynomial with only two elements to estimate the Volterra series. The mathematical function of this polynomial is expressed as follows:

(2) $$y=w_{0}+w_{1}x_{1}+w_{2}x_{2}+w_{3}x_{1}^{2}+w_{4}x_{2}^{2}+w_{5}x_{1}x_{2}$$

The main goal of the GMDH method is to identify the unknown parameters in the Volterra series by minimizing the sum of squared differences between the predicted and observed output values. These parameters, represented as $w_{i}$, are estimated through regression algorithms applied to each pair of input features, $x_{i}$ and $x_{j}$. This process enables the algorithm to iteratively refine and optimize the model by finding the most suitable coefficients that minimize the divergence between actual and predicted outcomes for the given input variables.

### 5.1 Dataset

Previous study results were utilized to estimate the compressive strength of FRC incorporating either steel or PP fibers. A comprehensive database was compiled, encompassing 286 distinct experimental records from the literature on the compressive strength of FRC. To further enrich the dataset and address gaps identified in existing research, 140 additional data points from recent experimental tests were also included. This expanded dataset provides a more robust foundation for analyzing and estimating the performance of FRC under various conditions. This dataset includes information on cement (C), water (W), coarse aggregates (CA), fine aggregates (FA), fiber content (F), fiber diameter (d), fiber length (l), fiber tensile strength (F_*t*_), fiber modulus of elasticity (E), heating rate (H), and maximum temperature (T).

The dataset also takes into account the age, mould size, and curing type of the tested specimens concerning relative compressive strength. Specifically, the compressive strength of each specimen at temperature *T* is compared with the same specimen at the same age, mould size, and curing conditions at ambient temperature. This approach allows for the consideration of the effects of age, curing, and mold size in the results. Table 6 presents statistical measurements, including maximum, minimum, mean, and standard deviation, for the input variables in concrete containing various fiber types, i.e., steel and PP fibers. These statistical parameters were calculated based on all 426 data points (286 from literature review and 140 from the current experimental tests) in the dataset. Among these parameters, standard deviation measures the amount of variation or dispersion of a random variable from its average value. For a standard deviation of 268 °C in temperature, this indicates that more than 68% of the data points fall within the range of 513 °C (average) ± 268 °C (standard deviation).

**Table 6:** Statistical measures for the concrete containing different fiber types (steel and PP).

Attribute	Unit	min	max	mean	standard deviation
Concrete mix design					

Water	*kg*/*m*^3^	143	233.28	173.90	27.34
Cement	*kg*/*m*^3^	290	600	452.43	73.12
Coarse agg.	*kg*/*m*^3^	151.78	1168	857.18	308.49
Fine agg.	*kg*/*m*^3^	540	1255	810.55	159.37

Mechanical and geometrical fiber characteristics					

Fiber content	*kg*/*m*^3^	0.452	118.5	28.15	33.63
Diameter	*mm*	0.007	2	0.46	0.39
Length	*mm*	1	65	28.09	19.16
Tensile strength	*MPa*	250	2800	931.37	669.78
Module of elasticity	*MPa*	1.6	240	96.06	98.32

Elevated temperatures characteristics					

Heating rate	°*C*/*min*	1	10	4.91	2.82
Max. temperature	°*C*	100	1200	513.39	268.45

**Figure 10:**
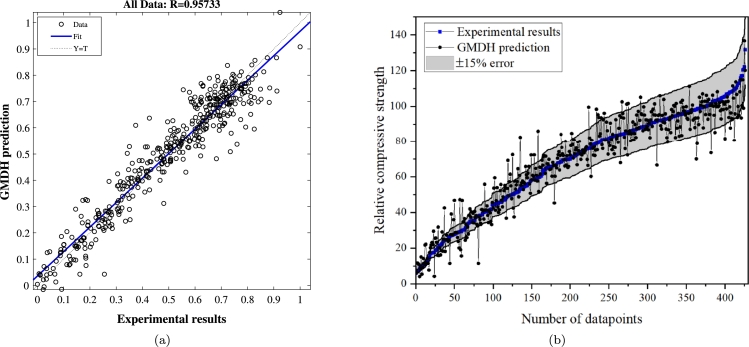
a) The regression curve, and b) comparing the experimental results with predictions.

The predictive performance of the GMDH technique for relative compressive strength is illustrated in Figs. 10(a) and 10(b). The results indicate satisfactory prediction quality, demonstrating the GMDH technique's success in forecasting relative compressive strength based on the input parameters. The proposed GMDH model is applicable to all concrete samples and mix designs within the ranges specified in Table 6. Given these parameters, it can be argued that most concrete mixtures can be effectively accommodated by the model. Additionally, the model can differentiate between fiber types based on mechanical properties such as tensile strength and modulus of elasticity. Thus, it is reasonable to assert that the model can handle various types of fibers used in concrete. It is noteworthy that other parameters, such as porosity and moisture content, could potentially influence the model. However, since these parameters were not measured or reported in most of the previously published papers, they cannot be included in the current model. Despite this, the accuracy of the proposed model remains promising, even without accounting for these factors, though their inclusion might further enhance the model's accuracy. Therefore, the limitation of the proposed model can be listed as (1) The model is designed to anticipate the relative compressive strength only within the specific range of data that was introduced during its development. This means that its accuracy and reliability may diminish when applied to data outside of this range, limiting its generalizability to other scenarios or datasets, and (2) The model primarily leverages the parameters mentioned earlier (such as those introduced in Table 6) to make its predictions. However, it does not account for other potentially influential factors that were not considered or measured.

The accuracy of the predicted relative compressive strength from the GMDH model was assessed using several statistical metrics, including mean squared error (MSE), root mean squared error (RMSE), mean absolute percentage error (MAPE), the Nash-Sutcliffe efficiency (NSE) coefficient, and the correlation coefficient (R). Equations (3) to (7) detail the computation of these metrics. Table 7 presents a comparison of these metrics across all data points generated by the GMDH model. An optimal value for statistical factors (excluding NSE and R) is zero, while for NSE and R, the ideal value is one. RMSE gauges the disparity between predicted and experimental values, while MAPE captures both prediction errors and their proportion to experimental values [68], [75]. NSE evaluates the model's predictive capability. The statistical indicators in Table 7 underscore the close proximity of predicted compressive strengths using the GMDH model to the test results that were conducted in the experiment, affirming the appropriateness of the suggested GMDH network.

(3) $$MSE=\frac{\sum {\left(\overset{ˆ}{y}-y\right)}^{2}}{N}$$

(4) $$RMSE=\sqrt{\frac{\sum {\left(\overset{ˆ}{y}-y\right)}^{2}}{N}}$$

(5) $$MAPE=\frac{100}{N}\sum |\frac{\overset{¯}{y}-y}{y}|$$

(6) $$NSE=1-\frac{\sum {\left(\overset{ˆ}{y}-y\right)}^{2}}{\sum {\left(\overset{¯}{y}-y\right)}^{2}}$$

(7) $$R=\frac{\sum (\overset{ˆ}{y}-\overset{ˆ}{\overset{¯}{y}})(y-\overset{¯}{y}))}{\sqrt{\sum {\left(\overset{ˆ}{y}-\overset{ˆ}{\overset{¯}{y}}\right)}^{2}}\sqrt{\sum {\left(y-\overset{¯}{y}\right)}^{2}}}$$

**Table 7:** Assessment of the GMDH network accuracy through the comparison of five statistical indicators.

Target	MSE	RMSE	MAPE	NSE	R
Relative compressive strength	0.0044	0.0666	17.45	0.9161	0.9573

### 5.2 Challenges

High-quality, labeled datasets with a wide range of concrete mix designs, curing conditions, heat rates, and temperatures are often scarce. The availability of diverse and comprehensive datasets is crucial for training robust models. The results of concrete compressive strength can be noisy, with outliers or errors in measurement especially when subjected to high temperatures that can negatively impact model training and predictions. Moreover, the relationship between input features and compressive strength is often non-linear and complex, requiring advanced techniques and models tuning to capture these relationships accurately. In addition, in fields like civil engineering, where safety and regulatory compliance are critical, the lack of interpretability in soft computing models can be a significant barrier to adoption.

## 6 Conclusion

The complex response of FRC at elevated temperatures can be analyzed through the application of soft computing methods. These approaches simultaneously take into account all relevant parameters and recognize their complex interrelationships. To support this analysis, an extensive dataset was compiled from prior studies and enriched through extensive experimental efforts. In the experimental study, the impact of varying quantities of cement consumption in commercially available ready-mix concrete, coupled with the presence of diverse types of fibers at different percentages, was empirically studied to assess concrete performance under elevated temperatures. A review of the literature revealed gaps in existing research. Additionally, an assessment of the spalling behavior and residual strength of concrete was conducted by analyzing the available data in the literature. The main results are as follows:

• The literature suggests that rising the steel fiber amount has a favorable impact on the compressive strength of FRC after being exposed to fire, particularly at elevated temperatures surpassing 300 °C. However, beyond this temperature, strength reduction occurred owing to uneven expansion and misalignment of shrinkage among the cement paste and aggregate. The enhanced strength can be associated to the bridging effect of steel fibers in the presence of cracks, along with the positive influence of steel fibers in bolstering the integrity of concrete during its deterioration phase.
• The existing literature does not provide a conclusive consensus on the effect of PP fiber amount on relative compressive strength. The notable discrepancies in the findings of previous researchers may arise from variations in other crucial factors. Therefore, it is imperative to take into account additional parameters such as aspect ratio, heating rate, concrete mix design, and other relevant factors to arrive at a definitive understanding.
• While higher heating rates can induce rapid and intense temperature increases within the concrete, potentially leading to thermal stresses and cracking, it becomes evident that the duration of the constant temperature period during which specimens are held plays a more pivotal role. As the rate of decomposition of calcium carbonate is gradual, an extension in duration of heating leads to increased decomposition, consequently causing a decline in strength at a later stage. Divergent opinions exist regarding the impact of heating rate on the relative compressive strength of FRC, with these variations suggested to be associated with early microcracks induced by the greater rate of heating.
• Increased heat exposure results in a reduction in relative compressive strength, with a more notable decline observed in specimens with lower W/C ratios when temperatures exceed 400 °C. Lower W/C ratios also correlate with a higher susceptibility to spalling at elevated temperatures. While concrete with lower W/C ratios performs better at temperatures below 400 °C, this advantage diminishes at higher temperatures due to factors such as a higher volume of aggregates, transformation of calcium hydroxide to calcium oxide, disintegration of C–S–H, and the impact of admixtures.
• At 7 days and room temperature, the inclusion of steel fibers to samples with cement contents of 350, 400, and 450 $kg/m^{3}$ resulted in average changes in compressive strength of +10.3%, -10%, and -17%, respectively. In contrast, PP fibers led to a reduction in the compressive strength by 13.7% and 2.6% on average at 7 and 28 days, respectively.
• Increasing the cement content reduces the efficiency of steel fibers in improving compressive strength at elevated temperatures. In contrast, incorporating PP fibers has been found to enhance the compressive strength at both 7 and 28 days.
• When the temperature reached 600 °C, some concrete specimens experienced spalling and explosive failure upon exiting the furnace. However, samples containing PP fibers did not undergo spalling, demonstrating a key advantage of these fibers in mitigating such failures.
• An increase in temperature generally reduces tensile strength. However, the inclusion of fibers to concrete specimens improves tensile strength. For instance, samples with 350 $kg/m^{3}$ of steel fibers at 200 °C show an average tensile strength enhancement of 68.2%. Moreover, a higher volume of fibers in the concrete correlates with a further increase in tensile strength.
• The five statistical indicators, MSE (0.0044), RMSE (0.0666), MAPE (17.45), NSE (0.9161), and R (0.9573), demonstrate a strong connection among the relative compressive strengths predicted by the GMDH network and the experimental results. These results confirm the efficiency of the GMDH model in accurately estimating the relative compressive strength of FRC incorporating various types of fiber.

## Funding

The authors did not receive support from any organization for the submitted work.

## CRediT authorship contribution statement

**M.S. Moradi:** Writing – review & editing, Writing – original draft, Visualization, Validation, Software, Methodology, Investigation, Formal analysis, Data curation. **M.H. Tavana:** Writing – review & editing, Supervision, Resources, Project administration, Funding acquisition. **M.R. Habibi:** Supervision. **M. Amiri:** Supervision.

## Declaration of Competing Interest

The authors declare that they have no known competing financial interests or personal relationships that could have appeared to influence the work reported in this paper.

## Data Availability

Data will be made available on request.
